# Host angiogenic reprogramming by *Echinococcus multilocularis* protoscoleces protein via PDGFR/PI3K/AKT cascade

**DOI:** 10.3389/fmicb.2025.1686956

**Published:** 2025-11-17

**Authors:** Xiaojuan Bi, Ning Yang, Ying Ke, Junlong Xue, Xue Zhang, Hui Liu, Jin Chu, Liang Li, Yingmei Shao, Guodong Lü, Tuerganaili Aji, Renyong Lin

**Affiliations:** 1 State Key Laboratory of Pathogenesis, Prevention, and Treatment of Central Asian High Incidence Diseases, Clinical Medical Research Institute, The First Affiliated Hospital of Xinjiang Medical University, Urumqi, Xinjiang, China; 2 Department of Hepatic Hydatid and Hepatobiliary Surgery, Digestive and Vascular Surgery Centre, The First Affiliated Hospital of Xinjiang Medical University, Urumqi, China

**Keywords:** *Echinococcus multilocularis*, pathological angiogenesis, human umbilical vein endothelial cells, PDGF-BB, PDGFR

## Abstract

**Background:**

Alveolar echinococcosis (AE) is a globally present zoonotic disease caused by *Echinococcus multilocularis* (*E. multilocularis*) infection, characterized by the formation of tumor-like growths primarily in the liver, with the potential to spread to other organs. Similar to tumors, *E. multilocularis* infection is accompanied by pathological angiogenesis, suggesting that the implementation of anti-angiogenic therapeutic strategies may also have promising applications in the treatment of AE. However, the mechanism of angiogenesis in AE remains unclear and has not been fully elucidated.

**Results:**

In this study, we discovered that angiogenesis related genes are significantly up-regulated in the mouse model of *E. multilocularis* infection and pathological angiogenesis around the lesion was significantly increased at 10–12 weeks after infection compared to the control group. Interventions utilizing a range of inhibitors at the *in vitro* level, including the PDGFR-β inhibitor AG1296, the PI3K inhibitor LY294002, the AKT inhibitor MK2206, and the FAK inhibitor Y15, demonstrated that *E. multilocularis* protoscoleces protein (EmP) induces angiogenesis through PDGFR/PI3K/AKT/FAK signaling pathway.

**Conclusion:**

Our findings provide new perspectives on how *E. multilocularis* infection triggers pathological angiogenesis in the host liver, and may provide a novel anti-angiogenic therapeutic strategy against *E. multilocularis* infection.

## Highlights

- Angiogenesis related genes are significantly up-regulated in the liver surrounding the lesion in *E. multilocularis* infected mice.
- *E. multilocularis* protoscoleces protein (EmP) induces pathological angiogenesis via the PDGFR/PI3K/AKT/FAK signaling pathway *in vitro*.

## 1 Introduction

Alveolar echinococcosis (AE) is a parasitic zoonotic disease distributed in both developing and developed Northern Hemisphere countries, with an estimated 17,400 new infections per year, most of which occur in China (Casulli et al., 2019). AE is caused by the larval stage (metacestode) of the tapeworm *E. multilocularis*. The eggs are ingested orally and hatch in the host's gastrointestinal tract, releasing an oncosphere which penetrates the epithelium in the small intestine and gains access to the inner organs, especially the liver. In the liver, the oncosphere undergoes metamorphosis into the metacestode. AE is characterized by the progressing infiltrative proliferation of the parasite, mimicking a malignancy (Herz and Brehm, 2021). The continuous growth of metacestodes erodes blood vessels and bile ducts in the liver tissue of intermediate hosts, leading to organ failure. Mortality in untreated or inadequately treated AE patients is more than 90% within 10 to 15 years of diagnosis (Yang et al., 2022). In recent years, significant progress has been made in the prevention, diagnosis and treatment of echinococcosis. However, considering the toxicity and inefficiency exhibited by presently accessible medications, insufficiency of surgical approaches, as well as the difficulties in controlling and preventing the condition, it is imperative to promptly explore novel therapeutic pathways and targets (Wen et al., 2019).

Large animals necessitate a circulatory system to facilitate the distribution of essential substances such as oxygen, nutrients, hormones, and growth factors throughout the entire body, while also facilitating the elimination of waste products including carbon dioxide, lactic acid, and urea. This vital function is carried out by the blood vascular system (Dennis et al., 2011). Similarly, parasites, as relatively large pathogens, they have high requirements comparable requirements in order to effectively establish themselves within their parasitized host. Throughout the development of various parasites that reside in tissues or blood vessels, it becomes evident that there are both direct and indirect interactions with the host's blood vascular systems (Dennis et al., 2011). To date, a variety of parasites have been identified that may have the ability to initiate or promote the growth of new blood vessels in the host (Andrade and Santana, 2010; El-Dardiry et al., 2021). Angiogenesis has been observed in the vicinity of lesions in canines and rodents infected with *E. multilocularis*. The emergence of these newly formed blood vessels may be important in the provision of nutritional factors, serves as potential mediators of signals governing host-parasite interactions, and potentially contributes to the development of metastasis (Weiss et al., 2010; Stadelmann et al., 2010; Perez Rodriguez et al., 2023). Nevertheless, the mechanism of angiogenesis caused by *E. multilocularis* infection remains unclear and has not been fully elucidated.

Pathological angiogenesis is intrinsically related to the progression of chronic liver diseases such as liver fibrosis and hepatocellular carcinoma (Bocca et al., 2015). Considering that angiogenesis is a crucial process for tumor growth and metastasis, the implementation of anti-angiogenic therapy represents a promising anti-cancer strategy with the objective of depriving tumors of essential nutrients and oxygen supply through the decrease of the vascular network and the avoidance of new blood vessels formation (Kountouras et al., 2005). As mentioned above, akin to tumors, parasitic infections are also commonly accompanied by pathological angiogenesis, especially in AE, also called “worm cancer.” Consequently, the implementation of anti-angiogenic therapeutic strategies may have promising therapeutic applications in the treatment of AE. In a recent study, Jiang et al. (2023) found that sunitinib malate (SU11248) treatment markedly reduced vascular endothelial growth factor A (VEGFA) induced angiogenesis and significantly inhibited the growth of multilocular tapeworms in *E. multilocularis* infected mice. This study preliminarily reveals the promise of anti-angiogenesis strategies in the treatment of AE.

Presently, the majority of anti-angiogenic agents approved for cancer treatment rely on targeting vascular endothelial growth factor (VEGF) actions, given that VEGF signaling is widely acknowledged as the principal promoter of angiogenesis (Lopes-Coelho et al., 2021). However, there are many pathways that regulate angiogenesis, and when one of these processes is blocked, others may play alternative roles, leading to the fact that therapeutic strategies targeting VEGF are not always effective (Vimalraj, 2022). Combining alternative effector factors that target the angiogenic pathway may be a potential solution for anti-angiogenic therapies. Malignant tumors including ovarian cancer (OC), non-small cell lung cancer (NSCLC), and hepatocellular carcinoma (HCC) are known to have tumor vascularization that is connected to over- stimulation of platelet-derived growth factor (PDGF) signaling, either alone or in conjunction with VEGFA (Levitzki, 2004). In AE patients, our previous study has demonstrated that the expression of PDGF-BB and platelet derived growth factor receptor beta (PDGFR-β) was significantly higher in the close liver tissue (CLT) compared to distant liver tissue (DLT) (Ke et al., 2022). In addition, serum PDGF-BB levels were significantly lower in patients with AE, especially in patients with high-activity lesions, compared with healthy subjects. This finding suggests that serum PDGF-BB levels may serve as a straightforward, non-invasive, and expeditious biomarker for evaluating the metabolic activity of lesions in AE patients (Ke et al., 2022). However, the underlying mechanisms of altered PDGF signaling expression in AE remain elusive.

In this study, we analyzed the role of PDGF signaling-mediated angiogenesis in *E. multilocularis* infected mice. We confirmed that pathological angiogenesis surrounding the lesion was observed at 10–12 weeks post-infection in *E. multilocularis* infected mice. Mechanistically, we found that *E. multilocularis* protoscoleces protein (EmP) induces angiogenesis through PDGFR/phosphatidylinositol 3-kinase (PI3K)/AKT Serine/Threonine Kinase (AKT)/Focal Adhesion Kinase (FAK) signaling pathway similar to PDGF-BB *in vitro*. Our study elucidated the underlying mechanism of pathological angiogenesis in AE, which is mediated by PDGF signaling. This finding lays a fundamental basis for the potential implementation of anti-angiogenic therapeutic approaches in the treatment of AE.

## 2 Materials and methods

### 2.1 Establishment of *E. multilocularis* infection models

Thirty female C57BL/6 mice (weighing about 18–20 g, Beijing Vital River Laboratory Animal Technology Co. Ltd, Beijing, China) were randomly assigned to five groups of five animals each: a sham-operated group (Sham group) and four groups representing different time points post-infection with *E. multilocularis* (2 weeks, 4 weeks, 10 weeks, and 12 weeks groups). Mice in *E. multilocularis* infection groups were inoculated with protoscoleces suspended in normal saline (3,000 PSCs/mouse in 200 μl normal saline) via the hepatic portal vein, as previously described (Yang et al., 2022), while mice in the sham operation group were injected with equivalent amount of normal saline. Briefly, protoscoleces were obtained from intraperitoneal lesions maintained in Mongolian gerbils, washed several times using PBS, counted under a microscope, and adjusted to the appropriate concentrations before injection. Mice were kept pathogen-free at 20–24 °C under a 12 h light/dark cycle. Before the experiment, mice had free access to a regular diet and water for 1 week. At the end of the infection period, mice were anesthetized with 4% isoflurane and maintained with 1% isoflurane (RWD, Shenzhen, Cat# 20072102). Before collecting the mouse samples, we euthanized the mice using an overdose of anesthetic. The animal experiments were approved by the Institutional regulations of Ethical Committee of the First Affiliated Hospital of Xinjiang Medical University (No. K202110-18). The study was carried out in compliance with ARRLVE guidelines.

### 2.2 Histopathological and immunohistochemical analyses

Liver samples were harvested from mice and divided into two parts, one part was processed for histopathological assessment while the other part was promptly cryopreserved in liquid nitrogen for RNA extraction. For histopathological analyses, samples were fixed in 4% paraformaldehyde for 48 h and embedded in paraffin. Hematoxylin & eosin (H&E), Periodic Acid-Schiff (PAS) and immunohistochemistry (IHC) staining were performed on serial sections (4 μm thick). In addition, liver fibrosis in mice was assessed using Sirius red staining as previously described (Yang et al., 2022). The H&E, PAS, Sirius red and IHC stained sections were imaged by light microscopy (BX43, Olympus, Japan), the result of Sirius red and IHC staining were quantitatively analyzed using the Image-Pro Plus software (Version 6.0.0.260, Media Cybernetics, USA). Differences in protein expression or fibrosis levels in each group were determined using the Average Optical Density (AOD = integrated optical density/area) of positive reactions. Three to five fields per section were randomly selected and analyzed at highpowerfield (400 × magnification). Antibodies used for immunohistochemical staining are presented in Supplementary Table S1.

### 2.3 Quantitative real-time PCR (qRT-PCR) analysis

Total RNA was extracted from liver tissues preserved in liquid nitrogen sing the TRIzol reagent (Invitrogen). Purified total RNA was quantified using Nanodrop ND2000 (NanoDrop Technologies, Wilmington, DE, USA) and reverse-transcribed using a PrimeScript RT reagent Kit (RR047A, Takara, Japan). Then, 2 μl of cDNA was mixed with 18 μl of Master Mix (miScript SYBR Green PCR Kit, 218073, Germany, Qiagen) after which RT-PCR was performed using the CFX96 Touch System (BioRad, Hercules, CA, USA). The 2–ΔΔCt method was used to calculate relative mRNA expressions which were normalized to the housekeeping gene *GAPDH* (human) or *ACTB* (mouse) (Yang et al., 2022). The primer sequences used for qRT-PCR are presented in Supplementary Table S2.

### 2.4 Cell culturing

In order to clarify whether the *E. multilocularis* has an angiogenesis-inducing effect *in vitro*, the human umbilical vein endothelial cells (HUVECs) cell line was purchased from the Cellverse Co., Ltd. (iCell-h110, Shanghai, China), and utilized this cell line for our *in vitro* experimentation. Cells were cultured in RPMI-1640 supplemented with 10% FBS and 1% Penicillin-Streptomycin (Gibco). The cells were grown in a humidified incubator at 37 °C with 5% CO_2_. The culture medium was refreshed every day.

### 2.5 *E. multilocularis* PSC protein (EmP) preparation and inhibitors intervention

EmP refers to the crude extract of proteins derived from *Echinococcus multilocularis* protoscoleces. For protein preparation, *E. multilocularis* PSC were collected from intraperitoneal lesions maintained in BALB/c mice. PSC were ruptured by sonication (30 s pulses of 30% power with 10 s intervals in triplicate). The lysates were left overnight on a rocker at 4 °C and centrifuged at 12,000 × g for 15 min at 4 °C. The supernatant was sterilely filtered (0.45 μm) and protein concentration was quantified using the BCA Protein Assay Kit (Thermo Fisher Scientific, USA). For *in vitro* experiments, EmP (5μg/ml) or recombinant human PDGF-BB (Peprotech, 50 ng/ml) were added into medium in the lower well of the transwell chambers. PDGFR-β inhibitor AG1296, PI3K inhibitor LY294002, Akt inhibitor MK2206, and FAK inhibitor Y15 were used under the standard protocols (Selleckchem, Germany). For *in vitro* experiments, HUVECs were pretreated with PDGFR-β inhibitor AG1296 (10 μM), PI3K inhibitor LY294002 (10 μM), Akt inhibitor MK2206 (10 μM) and FAK inhibitor Y15 (10 μM) were added into medium for 1 h, respectively. After inhibitor treatment, the cells were seeded in the upper well of the transwell chambers (three replicates per group).

### 2.6 Cell viability assay

HUVECs were seeded in 24 well plates (2 × 10^4^/well) and evaluated at 1–4 days after seeding. CCK8 solution (10 μl, Dojindo, Japan) was added to the medium per well according to the manufacturer's instructions and incubated with the cells for 3 h at 37 °C and 5% CO_2_. After incubation, the absorbance of the solution was measured at a wavelength of 450 nm (Multiskan MK3, Thermo, USA).

### 2.7 Transwell migration and tube formation assay

Transwell migration assay was performed in 96-well Transwell plates (Corning) with 8-μm pore filters. HUVECs were seeded in the upper chambers (2 × 10^4^/well) and then incubated with or without EmP or PDGF-BB in the lower chambers for 3 h. At the end of incubation, HUVECs were fixed with 10% formaldehyde and stained with crystal violet (Sigma-Aldrich) for 15 min sequentially. After gently washing three times by PBS for 5 min each time, cells that migrated through the membrane to the lower chambers were counted by light microscopy (BX43, Olympus, Japan) at 200 × magnification. Tube formation assay was performed in 96-well Transwell plates (Matrigel was polymerized 45 min at 37 °C). HUVECs were seeded in the upper chambers (2 × 10^4^/well) and then incubated with or without EmP or PDGF-BB in the lower chambers for 3 h. After incubation at 37 °C, pseudocapillary formation was pseudocapillary formation was imaged by light microscopy (BX43, Olympus, Japan). The parameters of pseudocapillary formation by HUVECs were measured by ImageJ with the Angiogenesis Analyzer plugin. The cumulative pseudocapillary length, the numbers of mesh and major junction were quantified in this study (Guan et al., 2020).

### 2.8 Statistical analysis

Statistical analyses were performed using the SPSS statistical software package version 20.0 (SPSS, Inc, Chicago, IL, USA) and GraphPad Prism (version 7.0d for MacOS X, USA, GraphPad Software, San Diego, California, USA). Significance was determined with the two-tailed Student's *t*-test for comparison of two groups. Significance between multiple groups was determined by one-way ANOVA followed by Tukey's *post hoc* test. ^*^P < 0.05 was the threshold for significance. Error bars represent ± standard error of the mean (SEM).

## 3 Results

### 3.1 Increased expression of PDGF-BB/PDGFR-β and angiogenesis around liver lesions in *Echinococcosis multilocularis* infected mice

In our prior investigation, it was observed that the mRNA and protein expression levels of PDGF-BB and PDGFR-β exhibited a noteworthy increase in the close to the lesion (CLT) group when compared to the distal to the lesion (DLT) group among AE patients (Ke et al., 2022). The PDGF family is composed of polypeptide homodimers of PDGF-A, B, C, and D, as well as the heterodimer PDGF-AB. These ligands bind to PDGFR and tyrosine kinase receptors, triggering pathways that exhibit similarities or identical characteristics to those induced by VEGF. Activation of PDGF signaling is associated with growth, survival and angiogenesis of various cells (Su et al., 2020). Therefore, we hypothesized that PDGF-BB is involved in hepatic angiogenesis in alveolar echinococcosis and proceeded to conduct an investigation on this matter.

The *E. multilocularis* infection mouse model was established to investigate the mechanism of angiogenesis in alveolar echinococcosis. Animals were sacrificed at 2-, 4-, 10- and 12-weeks post-infection, respectively. With the prolongation of infection, the lesion volume gradually increased and normal liver tissue is constantly being eroded (Supplementary Figure S1A). PAS staining showed the laminated layer of the metacestode (Supplementary Figure S1B). The results of Sirius red staining and α-SMA immunohistochemical staining demonstrated a progressive increase in the extent of fibrosis as the duration of infection prolonged. Additionally, significant destruction of the hepatic architecture was observed, particularly during the later stages of infection (10 to 12 weeks) (Supplementary Figures S1C–F).

**Figure 1:**
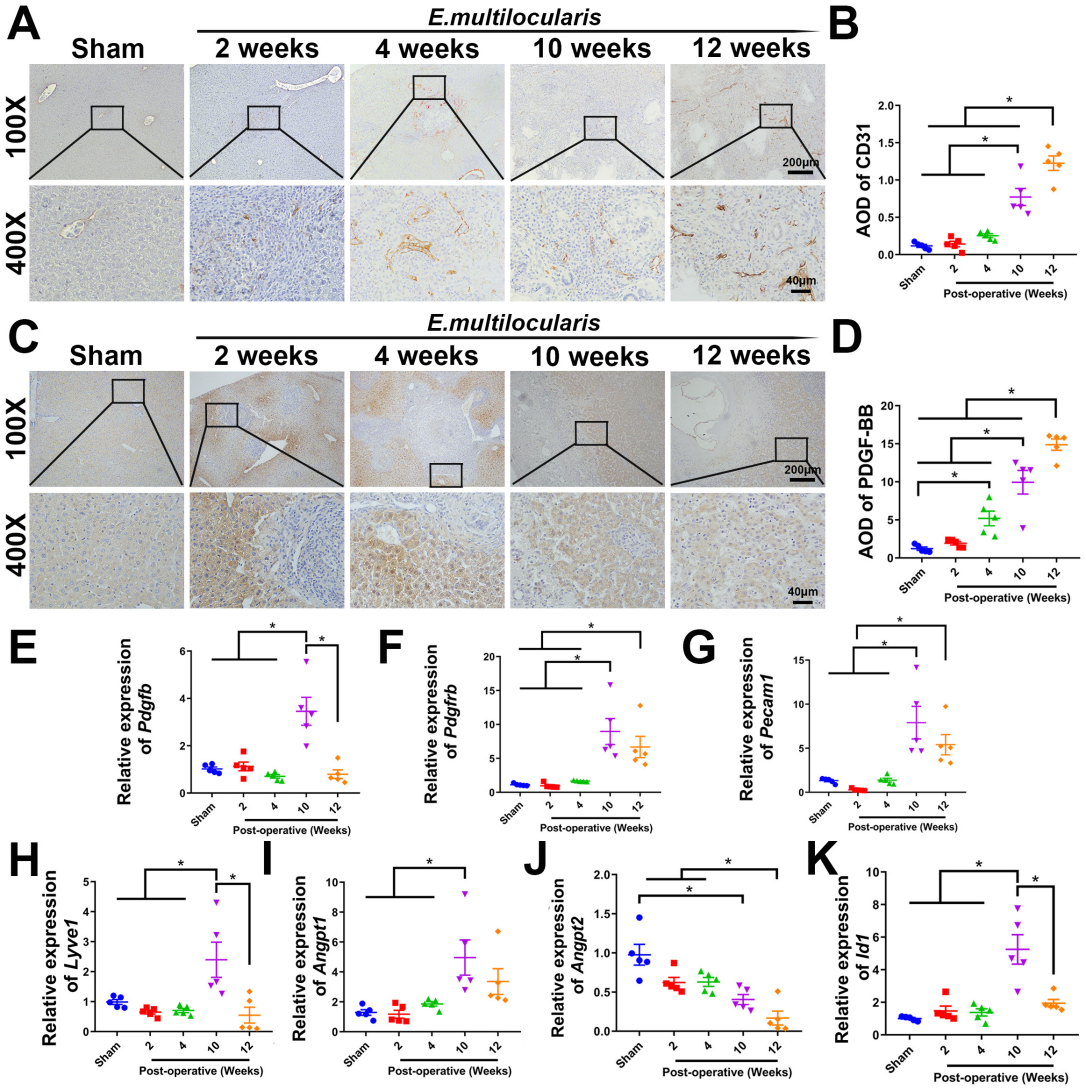
Increased hepatic angiogenesis in *E. multilocularis* infected mice. **(A, B)** Immunohistochemical staining of CD31. **(C, D)** Immunohistochemical staining of PDGF-BB. Quantitative real-time PCR detection of *Pdgfb*. **(E, F)** Quantitative real-time PCR detection of *Pdgfb* and *Pdgfrb*. **(G–K)** Quantitative real-time PCR detection of *Pecam1, Lyve1, Angpt1, Angpt2*, and *Id1* genes expression. All data are presented as mean ± SEM. *P < 0.05, *n* = 5.

To clarify whether angiogenesis was present in the livers of *E. multilocularis* infected mice, we examined the expression of angiogenesis-related genes in the liver. A notable augmentation in angiogenesis surrounding the lesion was observed at 10–12 weeks post-infection, as evidenced by immunohistochemical staining of CD31, in contrast to the sham operation group. In addition, the angiogenesis observed at 2–4 weeks post-infection was also increased in comparison to the sham operation group, but there was no statistical significance (Figures 1A, B). Furthermore, the result of qRT-PCR analysis and immunohistochemical staining showed that the expression of PDGF-BB and PDGFR-β were significantly elevated at 10–12 weeks post-infection (Figures 1C–F). qRT-PCR analysis also showed that the angiogenesis-related genes, Angpt1 (Ezaki et al., 2024), Id1 (Guo et al., 2023), and Lyve1 (Lu et al., 2023), were significantly upregulated at 10 weeks post-infection, and Pecam1 (Zhang et al., 2024) was significantly upregulated at 10–12 weeks post-infection. As an antagonistic gene of *Angpt1*, the mRNA level of Angpt2 (Ezaki et al., 2024) was significantly down-regulated at 10–12 weeks post-infection, indicating elevated angiogenesis (Figures 1G–K).

### 3.2 EmP exhibits a PDGF-like pro-angiogenic function *in vitro*

**Figure 2:**
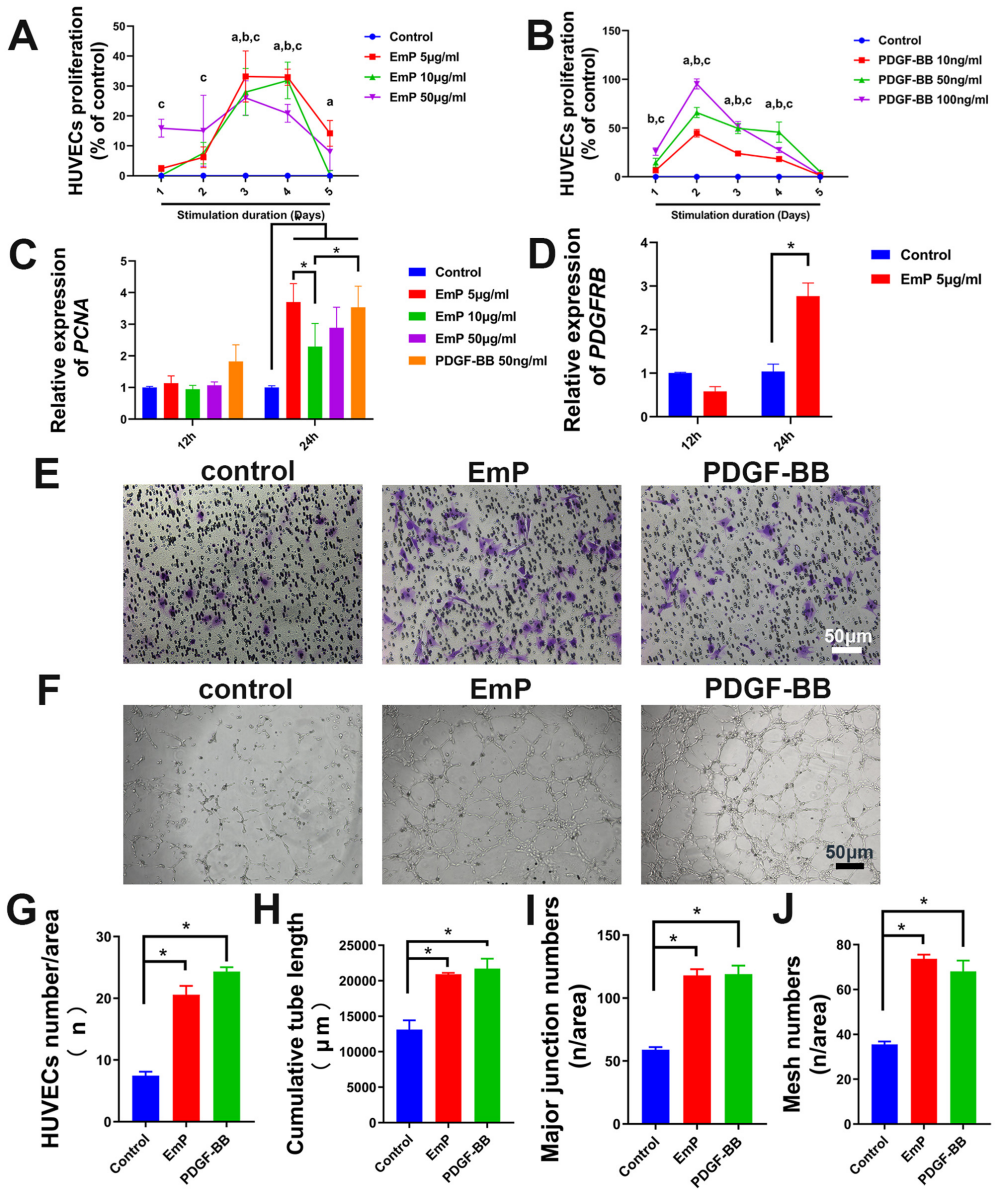
EmP significantly enhances the pseudocapillary formation ability of HUVECs in vitro. **(A)** Effect of EmP on the proliferation of HUVECs detected by CCK8 assay. ^*a*^*P* < 0.05, Emp 5 ug/ml compared with Control, ^*b*^*P* < 0.05, Emp 10 ug/ml compared with Control, ^*c*^*P* < 0.05, Emp 15 ug/ml compared with Control. **(B)** Effect of PDGF-BB on the proliferation of HUVECs detected by CCK8 assay. ^*a*^*P* < 0.05, PDGF-BB 10 ng/ml compared with Control, ^*b*^*P* < 0.05, PDGF-BB 50 ng/ml compared with Control, ^*c*^*P* < 0.05, PDGF-BB 100 ng/ml compared with Control. **(C)** Quantitative real-time PCR detection of PCNA after EmP and PDGF-BB stimulation. **(D)** Quantitative real-time PCR detection of PDGFRB after 679 EmP stimulation. **(E)** Representative images of transwell migration assay. **(F)** Representative images of pseudocapillary formation assay. **(G)** HUVECs counting results of transwell migration assay. **(H)** Counting results of the cumulative pseudocapillary length in HUVECs cells. **(I)** Counting results of the major junction numbers in HUVECs cells. **(J)** Counting results of the mesh numbers in HUVECs cells. Experiments were repeated three times and all data are presented as mean ± SEM. *P < 0.05. *n* = counts.

The results of CCK-8 assay showed that the proliferative ability of HUVECs was significantly enhanced after treatment with PDGF-BB or EmP, where PDGF-BB showed dose-dependent within a certain range. Specifically, the proliferation of HUVECs was significantly increased when treated with EmP at 5 μg/ml or PDGF-BB at 50 ng/ml (Figures 2A, B). In addition, the expression of proliferating cell nuclear antigen (PCNA) also demonstrated that stimulation with 5 μg/ml EmP for 24 h significantly promoted the proliferation of HUVECs (Figure 2C). Interestingly, we found that EmP at 5 μg/ml can promote the expression of PDGFR-β in HUVECs cells like PDGF-BB, which may partly explain the pro-angiogenic function of EmP (Figure 2D).

To further evaluate the angiogenic effects of EmP on HUVECs, we conducted the migration assay and tube formation assay. The migration assay displayed that the migratory ability of the HUVECs in the both PDGF-BB-treatment or EmP treatment groups were significantly enhanced compared with the control group (Figures 2E, F). The tube formation assay showed that the EmP, functions similar to PDGF-BB, significantly increased the cumulative tube length, major junction numbers and mesh numbers in HUVECs cells, indicating that EmP can significantly enhanced the tube formation ability of HUVECs *in vitro* (Figures 2G–J).

### 3.3 EmP induces angiogenesis through PDGFR/PI3K/AKT/FAK signaling pathway similar to PDGF-BB

**Figure 3:**
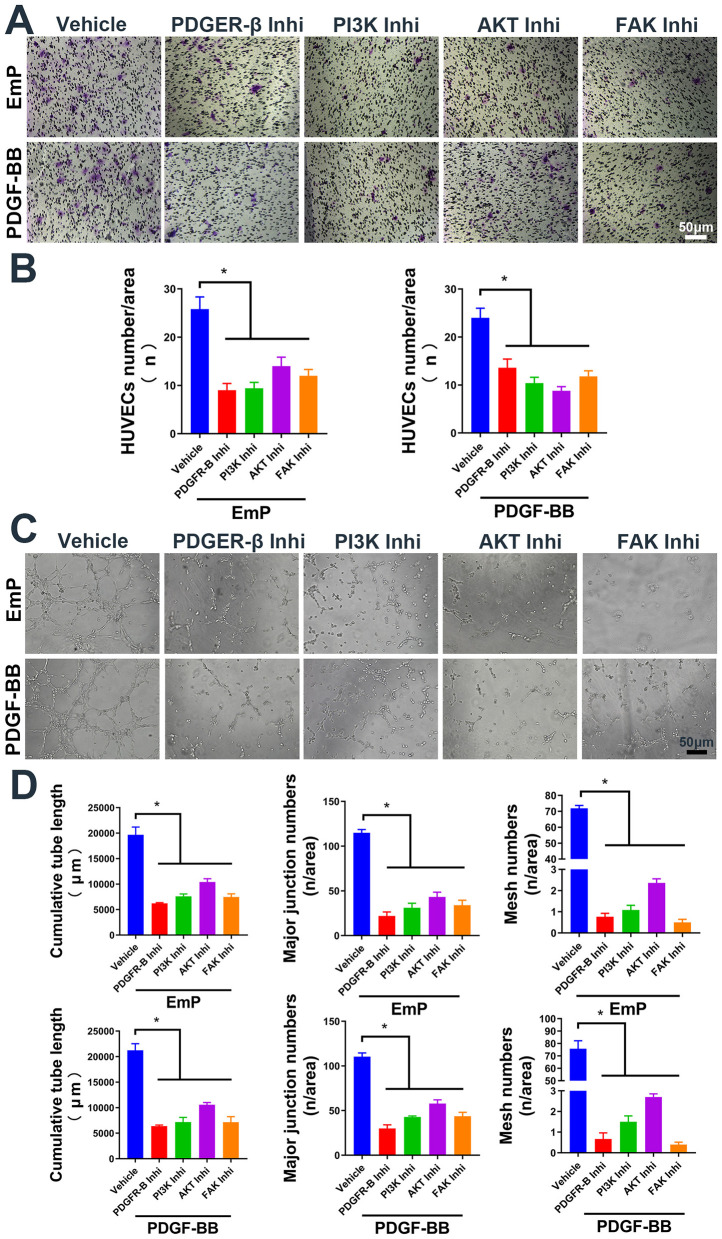
EmP promote HUVECs migration and pseudocapillary formation through PDGFR/PI3K/AKT/FAK pathway. **(A)** Representative images of transwell migration assay in HUVECs with inhibitor intervention. **(B)** HUVECs counting results of transwell migration assay with inhibitor intervention. **(C)** Representative images of pseudocapillary formation assay in HUVECs with inhibitor intervention. **(D)** Counting results of the cumulative pseudocapillary length, major junction numbers and mesh numbers in HUVECs with inhibitor intervention and co-treatment with EmP or PDGF-BB. Experiments were repeated three times and all data are presented as mean ± SEM. *P < 0.05. Inhi, inhibitor.

**Figure 4:**
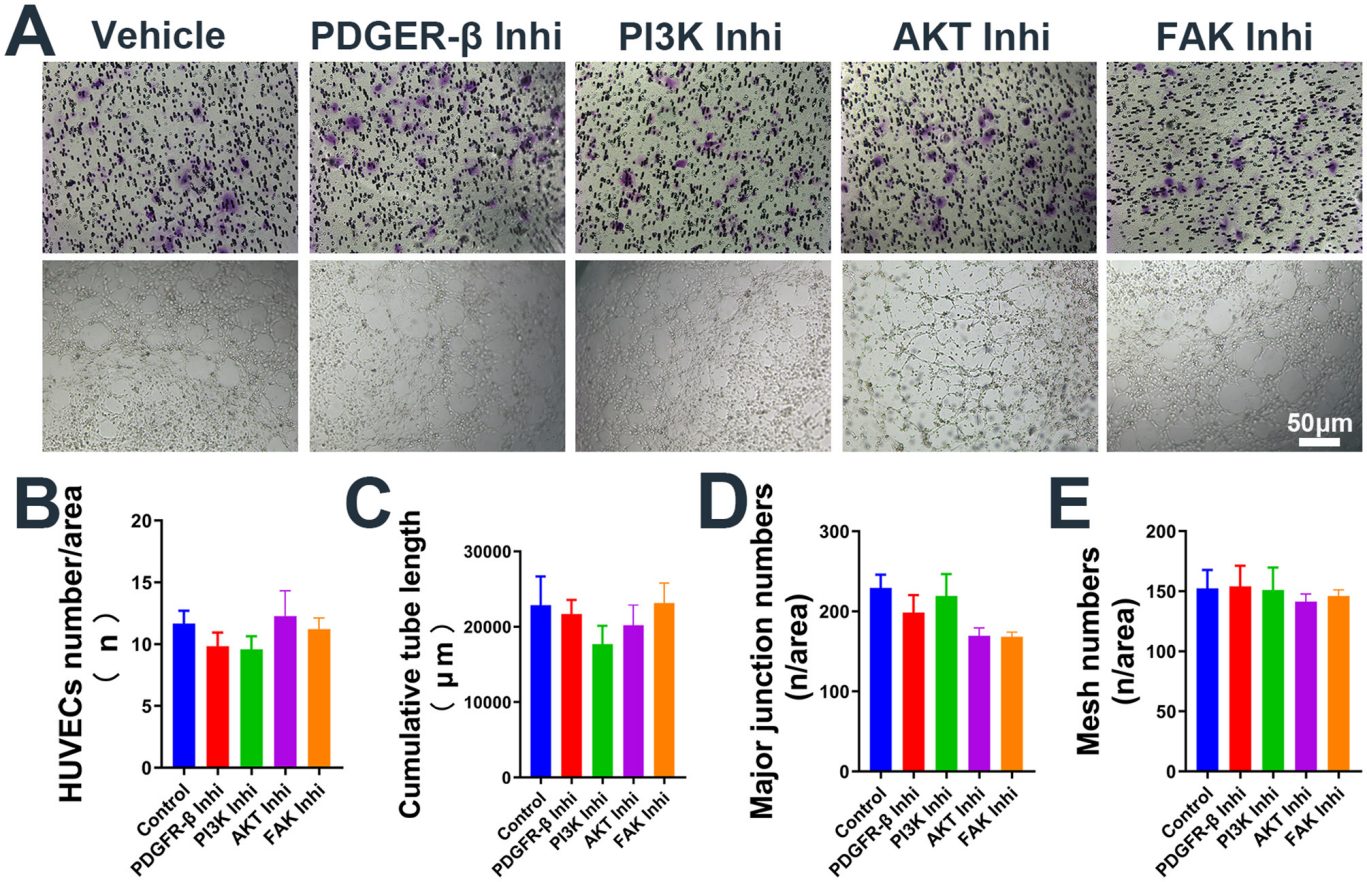
Administration of inhibitors alone the use of inhibitors alone does not affect the migration and pseudocapillary formation of HUVECs. **(A)** Representative images of transwell migration assay (Top) and pseudocapillary formation assay (Bottom) in HUVECs with inhibitor alone. **(B)** HUVECs counting results of transwell migration assay with inhibitor alone. **(C)** Counting results of the cumulative pseudocapillary length in HUVECs with inhibitor alone. **(D)** Counting results of the major junction numbers in HUVECs with inhibitor alone. **(E)** Counting results of the mesh numbers in HUVECs with inhibitor alone. Experiments were repeated three times and all data are presented as mean ± SEM. Inhi, inhibitor. n = counts.

**Figure 5:**
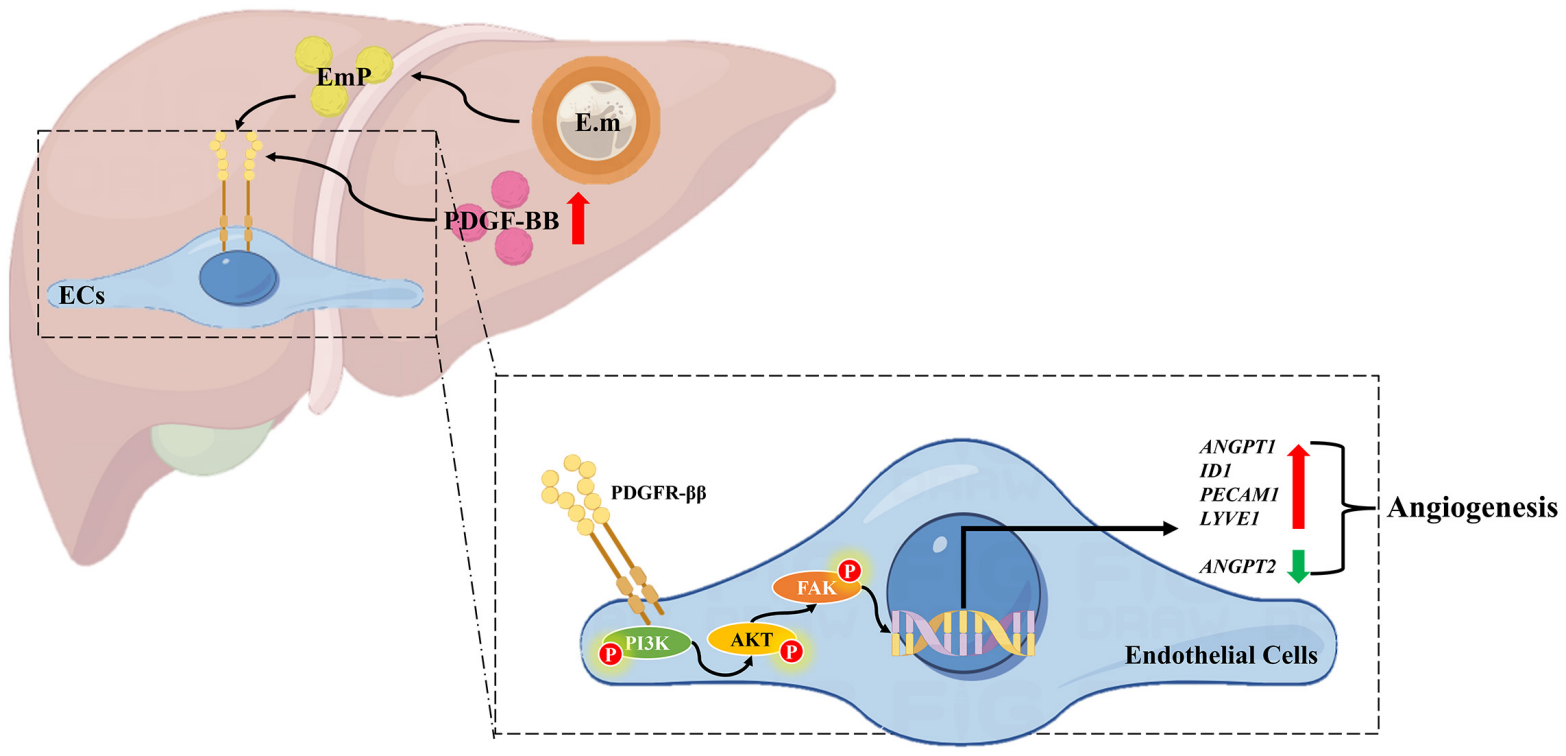
Schematic diagram of pro-angiogenesis mechanism in *Echinococcus multilocularis* infection. On the one hand, *Echinococcus multilocularis* infection directly upregulates PDGF-BB expression in the host liver, thereby stimulating angiogenesis through the activation of the PDGFR/PI3K/AKT/FAK signaling pathway. On the other hand, the secretion of EmP also contributes to angiogenesis by acting on the PDGFR/PI3K/AKT/FAK signaling pathway. The pathological angiogenesis in the peri-lesion area provides nutrients for the growth and development of the parasite, thus facilitating parasitism. E.m, *E. multilocularis* infection; EmP, *E. multilocularis* protoscoleces protein.

To further investigate the mechanism by which EmP promotes angiogenesis, we cultured HUVECs with or without EmP and examined PI3K signaling pathways, which known to play an important role in cell migration, cell growth, and invasion (Glaviano et al., 2023). We intervened the phosphorylation of PDGFR-β and other downstream kinases on HUVECs using inhibitors, and the results showed that independent inhibition of each component of PDGFR signaling pathway, was sufficient to block EmP or PDGF-BB induced HUVECs migration and pseudocapillary formation (Figures 3A–D). In addition, administration of inhibitors alone did not significantly affect the migration and pseudocapillary formation of HUVECs and there were no statistical differences between groups (Figures 4A–E). Taken together, the results suggest that EmP induced angiogenesis is dependent on the PDGFR/PI3K/AKT/FAK signaling pathway similar to PDGF-BB (Figure 5).

## 4 Discussion

Accumulating evidence indicates that parasites can increase survival within host by regulating host's angiogenesis. In *Schistosoma mansoni* (*S. mansoni*), the angiogenesis is induced by inflammation or released ovular antigens, which promote the activation and proliferation of endothelial cells (Francisco et al., 2022). Specifically, in *S. mansoni* infected mice, serum VEGF levels and the expression of Heme oxygenase-1 were progressively increased, which may serve as new indicators of progression of *S. mansoni* associated angiogenesis (Huwait et al., 2021). Bevacizumab has a promising protective effect against the *Schistosoma* induced angiogenesis. A regression in the vascular activity and microvascular density was observed in the infected mice following the administration of bevacizumab (Hasby Saad and El-Anwar, 2020). Furthermore, Paeoniflorin, an herbal extract derived from paeony, exhibits anti-angiogenic effects and also significantly reduces the mean egg count/gram stool, worm burden and egg count/gram liver tissue in *S. mansoni* infected mice (Abd El-Aal and Abdelbary, 2019). In trichinellosis, the *Trichinella spiralis* (*T. spiralis*) is capable of inducing angiogenesis and attracting highly permeable blood vessels to the surface of its collagenous capsule. This process facilitates the acquisition of nutrients and disposal of waste, thereby sustaining a prolonged host-parasite association (Rayia et al., 2022). Treatment with bevacizumab exhibited anti-angiogenic activities during the muscular phase of infection through down-regulating the expression of VEGF and CD31 (Rayia et al., 2022; Fadil et al., 2022). Similarity, zinc oxide nanoparticles decrease adult counts in the intestine and larval deposition in muscle through suppress the expression of VEGF induced by *T. spiralis* (Ashoush and Soliman, 2023). Unexceptionally, therapeutic strategies based on anti-angiogenesis strategies have also been applied to a variety of parasitic diseases such as chagas disease (Vellasco et al., 2022), malaria (Park et al., 2019), heartworm disease (Zueva et al., 2020; Machado et al., 2023), and neurocysticercosis (Carmen-Orozco et al., 2019), etc. Based on the aforementioned information, we pose the following scientific questions: Does echinococcosis exhibit similarities on angiogenesis to the aforementioned parasitic diseases? Can anti-angiogenesis strategies be equally applicable in the treatment of echinococcosis?

With the development of bioinformatics, multi-omics technology has been gradually applied in the study of echinococcosis. Consistent with our study, it has been demonstrated that the invasive growth of *E. multilocularis* is accompanied by notable angiogenesis and the expression of CD31 (Qing et al., 2019). Besides, through transcriptomic analysis, Yimingjiang et al. (2023) similarly found that angiogenesis-related genes *ADAM12, APLN, TWIST1, SPP1, RSPO3*, and *FOXC2* were elevated in the CLT group. The proteomic analysis revealed exosomes derived from *E. multilocularis* protoscoleces can be involved in focal adhesion and potentially promote angiogenesis. Increased angiogenesis was observed and the expression of several angiogenesis-regulated molecules, including VEGF, MMP9, MCP-1, SDF-1, and serpin E1 were increased in livers of *E. multilocularis* infected mice (Liu et al., 2023). These results suggest that angiogenesis is a possible mechanism underlying the tumor-like biological behavior observed during *E multilocularis* infection. Clinically, CT perfusion imaging with microvessels density reflected different situation of angiogenesis around lesions, indicating the presence of blood perfusion at the periphery of the lesion (Wang et al., 2011). All of the above studies suggest that anti-angiogenic therapeutic strategies are also promising in the treatment of AE. However, further validation of angiogenesis-regulated genes expression in the liver of AE patients is needed to clarify the reliability of therapeutic targets.

In many pathological conditions, developmental angiogenic processes are recapitulated, including but not limited to atherosclerosis, arthritis, psoriasis, endometriosis, and obesity, especially in various tumors. Thus, pathological angiogenesis presents new challenges yet new opportunities for the design of vascular-directed therapies (Dudley and Griffioen, 2023). Currently, the majority of approved agents for anti-angiogenic treatment primarily focus on the modulation of VEGF effects. Bevacizumab, the first VEGF-targeted agent approved by the Food and Drug Administration for cancer treatment, has been made accessible for cancer therapy (Kopetz et al., 2010). In *E. multilocularis* infection, elevated expression of VEGFA and VEGFR2 in the liver may mediate angiogenesis in *Echinococcus multilocularis* infection (Jiang et al., 2020). Furthermore, the administration of SU11248 markedly reduced neovascular formation and substantially inhibited *E. multilocularis* metacestode growth in mice. Moreover, SU11248 treatment also reduced the expression of VEGFA, VEGFR2, and p-VEGFR2 significantly (Jiang et al., 2023). However, compensatory mechanisms of other angiogenic mediators may be account for the resistance observed in patients when the VEGF signaling pathway is blocked. Therefore, targeting multiple effectors of angiogenic pathways simultaneously could potentially be an effective approach for anti-angiogenic therapies (Lopes-Coelho et al., 2021).

In addition to VEGF, PDGF-BB is a chemotactic and mitogenic factor within the PDGF family, which plays a crucial role in facilitating the migration, proliferation, and differentiation of diverse mesenchymal cell populations, including endothelial progenitor cells and mesenchymal stem cells, thereby promoting the processes of angiogenesis and osteogenesis (Andrae et al., 2008; Peng et al., 2020). PDGFs and their corresponding receptors, PDGFRα and PDGFRβ, exhibit expression in a diverse range of malignant tumors, such as non-small cell lung cancer (NSCLC), gastrointestinal stromal tumor (GIST), pancreatic cancer, breast cancer, ovarian carcinoma and hepatocellular carcinoma (Pandey et al., 2023). The findings obtained from employing various antibodies and inhibitors, including MOR8457 (Kuai et al., 2015), 1-NaPP1 (Tsioumpekou et al., 2020), CP-673451 (Yang et al., 2018), among others (Zou et al., 2022), indicate that targeting the PDGF/PDGFR signaling pathway represents a promising strategy for the treatment of cancer via regulating blood vessel regeneration. During osteoarthritis development, it was observed that the expression of PDGFR-β, primarily in the CD31^hi^Emcn^hi^ endothelium, was significantly increased in subchondral bones obtained from patients with osteoarthritis as well as in rodent models. PDGFR-β deletion specifically in endothelial cells reduced the number of H-type vessels, improvement in subchondral bone degradation, and mitigation of cartilage degeneration (Cui et al., 2022). Furthermore, PDGFR-β was increased in methionine-choline-deficient and high-fat (MCDHF)-induced liver injury in mice with a positive correlation to fibrosis and angiogenesis-related genes. The inhibition of liver fibrogenesis and angiogenesis is achieved by miR-26b-5p through its direct targeting of PDGFR-β, which may represent an effective therapeutic strategy for liver fibrosis (Yang et al., 2019). Our previous study also confirmed that the mRNA and protein expression levels of PDGF-BB and PDGFR-β exhibited a noteworthy increase in the CLT group when compared to the DLT group in AE patients (Ke et al., 2022). In this study, we further demonstrated that the expression of both PDGF-BB and PDGFR-β were significantly upregulated in *E. multilocularis* infected mice. The use of PDGFR-β inhibitor can block the pro-angiogenesis effect of EmP and PDGF-BB in HUVECs. These results suggest that PDGF-BB/PDGFR signaling are involved in the host's pathological angiogenesis, and blocking this signaling is expected to be a new approach for the treatment of AE.

The activation of the PDGF/PDGFR signaling pathway has been observed to be associated with angiogenesis through the regulation of multiple downstream pathways, such as PI3K/AKT pathway and Notch pathway (Zou et al., 2022). The activation of PI3K/AKT pathway plays a pivotal role in enhancing tumor angiogenesis, as evidenced by the numerous anti-vascular regeneration drugs that have been developed to specifically target this signaling pathway (Liu et al., 2019). Studies have demonstrated a significant positive correlation between the expression level of PI3K/AKT pathway and the formation of endothelial cell proliferation and survival in angiogenic experiments conducted using roxarsone (Zhu et al., 2013; Wang et al., 2016). In addition, in ischemic stroke rats, catalpol treatment significantly increased the expression of VEGF through up-regulating PI3K/AKT signaling and subsequently increasing FAK and Paxillin (Wang et al., 2022). Here, we provide evidence that EmP or PDGF-BB can induce phosphorylation of PI3K/AKT through up-regulating PDGFR-β, subsequently facilitating cell migration and pseudocapillary formation in HUVECs. The administration of PI3K/AKT inhibitors effectively impeded this process. FAK, a non-receptor protein tyrosine kinase, has been observed to be overexpressed in various types of cancer cells and peripheral vascular cells of solid tumors (Shiau et al., 2021; Arora et al., 2022). Previous studies have indicated that FAK plays a role in regulating tumor angiogenesis (Shiau et al., 2021; Zhang et al., 2023; Cai et al., 2019). In this study, the application of FAK inhibitors has been observed to impede the pro-angiogenesis impact of EmP and PDGF-BB, while having no discernible impact on the activation status of the PI3K/AKT pathway. This suggests that FAK, as a downstream element of the PI3K/AKT pathway, plays a role in promoting angiogenic activity. Taken together, these results suggest that blocking the PI3K/AKT/FAK pathway has the potential to inhibit angiogenesis and may be an effective treatment for AE patients.

However, many questions remain unanswered. Firstly, the angiogenesis-related genes, which were upregulated in *E. multilocularis* infected mice needs to be further verified in AE patients. Our previous study only demonstrated that PDGF-BB and PDGFR-β were highly expressed in the CLT group of AE patients, and validation of other angiogenesis-related genes will be our next move. Secondly, we found in this study that EmP has a pro-angiogenesis regenerative effect and mechanism similar to that of PDGF-BB, whether it is because *E. multilocularis* itself secretes PDGF-BB-like protein. This has not been clearly demonstrated by existing studies and needs to be confirmed by further studies on *E. multilocularis* secretome (Huang et al., 2017; Wang et al., 2015; Ahn et al., 2015, 2017). Thirdly, in the present study we performed a series of signaling pathway blockades on HUVECs *in vitro*, and preliminarily demonstrated that blocking the PDGFR/PI3K/AKT/FAK pathways could inhibit pathological angiogenesis. However, whether blockade of this pathway is equally effective *in vivo* is still unknown and needs to be confirmed by further studies.

## 5 Conclusion

In conclusion, our study confirms that the increased expression of PDGF-BB and PDGFR-β in *E. multilocularis* infected mice is associated with peri-lesion pathological angiogenesis. EmP has the capability to stimulate angiogenesis through the PDGFR/PI3K/AKT/FAK signaling pathway *in vitro*. The results of our study indicate that inhibiting PDGF signaling could potentially serve as an effective anti-angiogenic therapeutic strategy for the treatment of AE patients.

## Data Availability

The original contributions presented in the study are included in the article/Supplementary material, further inquiries can be directed to the corresponding authors.
