# Supplementary material for: Host angiogenic reprogramming by Echinococcus multilocularis protoscoleces protein via PDGFR/PI3K/AKT cascade
Source: Front Microbiol. 2025 Nov 17;16:1686956. doi: 10.3389/fmicb.2025.1686956 (PMC12665720; doi:10.3389/fmicb.2025.1686956)
Supplement: Supplementary file 1 [file Data_Sheet_1.docx]

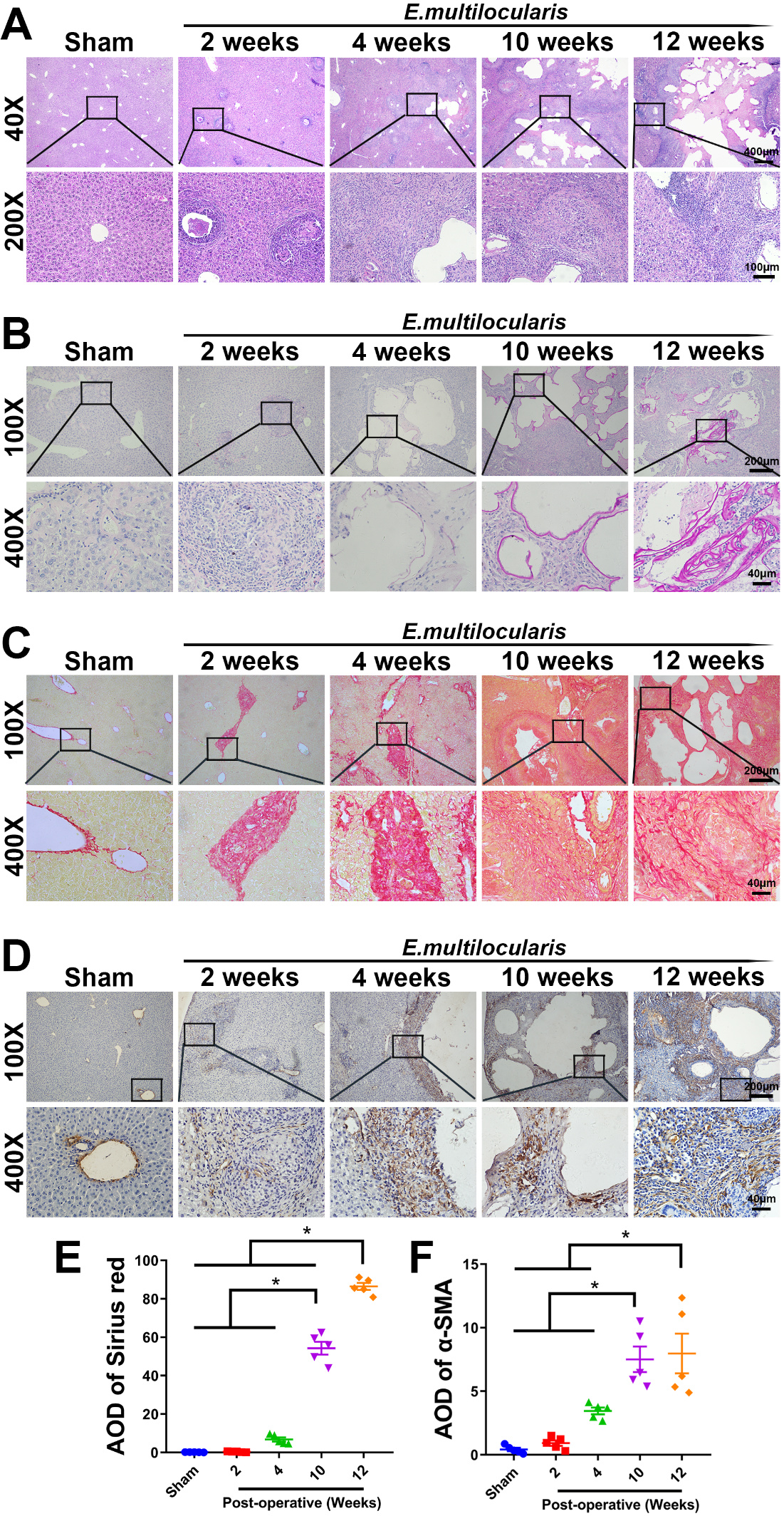


**S1 Fig. The hepatic histopathological alterations in *E. multilocularis* infected mice.** The pathological changes in the liver samples were evaluated by histological and immunohistochemical analyses. **A.** H&E staining of the liver sections. **B.** PAS staining of the liver sections. **C, E.** Sirius Red staining of the liver sections. **D, F.** Immunohistochemical staining of α-SMA. All data are presented as mean ± SEM. **P*＜0.05.

**S1 Table.** Antibodies information used in this study.

| **Target** | **Description** | **Application** | **Catalog number** | **Supplier** |
| --- | --- | --- | --- | --- |
| Anti-alpha-SMA | Rabbit monoclonal | IHC | ab124964 | Abcam |
| Anti-CD31 | Rabbit monoclonal | IHC | 77699 | CST |
| Anti-PDGFB | Rabbit polyclonal | IHC | ab23914 | Abcam |
| HRP secondary antibody | Goat Anti-rabbit IgG (H+L) | IHC | Ab6013 | Abcam |

**IHC, Immunohistochemistry.**

**S2 Table.** Sequence information for primers used in the study.

| **Gene** | **Species** | **Forward primer** | **Reverse primer** |
| --- | --- | --- | --- |
| *GAPDH* | human | GCACCGTCAAGGCTGAGAAC | TGGTGAAGACGCCAGTGGA |
| *PCNA* | human | GAAGGTGTTGGAGGCACTCAAGG | GCAGCGGTAGGTGTCGAAGC |
| *PDGFRB* | human | TGTGAATGACCATCAGGATGAA | CAGCTCAGCAAATTGTAGTGTG |
| *Actb* | mouse | TCGGATACTTCAGCGTCAGGA | GTCCCAGACATCAGGGAGTAA |
| *Pdgfb* | mouse | GCCTGCTGTAATCGCCGAGTG | GCGGCCACACCAGGAAGTTG |
| *Pdgfrb* | mouse | CACCTTCTTGCAGCGACACTCC | TCCATGTAGCCACCGTCACTCTC |
| *Pecam1* | mouse | TCCAATGACAACCACCGCAATGAG | AGCCAGCAGTATGAGGACCAGTC |
| *Angpt1* | mouse | CACAGGGACAGCAGGCAAACAG | CACAGGCATCGAACCACCAACC |
| *Angpt2* | mouse | CCAGGTCACCACTTGCACACAC | CAGGCCAGCCATTCTCACAGC |
| *Id1* | mouse | ACAGCGGGCGAGGTGGTAC | GCAGCCGTTCATGTCGTAGAGC |
| *ve1* | mouse | GCCACAACTCATCCGACACCTG | CAAGTAGGCGCTGCTGCTGAC |
